# Multi-cohort study on cytokine and chemokine profiles in the progression of COVID-19

**DOI:** 10.1038/s41598-024-61133-z

**Published:** 2024-05-06

**Authors:** Chaolin Huang, Xujuan Hu, Delong Wang, Rui Gong, Qiongya Wang, Fuli Ren, Yuanjun Wu, Juan Chen, Xianglian Xiong, Huadong Li, Qian Wang, Gangyu Long, Dingyu Zhang, Yang Han

**Affiliations:** 1 Center for Translational Medicine, The Eighth Clinical College, Tongji Medical College of Huazhong University of Science and Technology, Wuhan, 430023 Hubei China; 2 State Key Laboratory for Diagnosis and Treatment of Severe Zoonotic Infectious Diseases, Wuhan, 430023 Hubei China; 3 Division of Life Sciences and Medicine, The First Affiliated Hospital of University of Science and Technology of China, USTC, Hefei, 230001 Anhui China

**Keywords:** Biomarkers, Diagnostic markers, Predictive markers, Prognostic markers

## Abstract

Various substances in the blood plasma serve as prognostic indicators of the progression of COVID-19. Consequently, multi-omics studies, such as proteomic and metabolomics, are ongoing to identify accurate biomarkers. Cytokines and chemokines, which are crucial components of immune and inflammatory responses, play pivotal roles in the transition from mild to severe illness. To determine the relationship between plasma cytokines and the progression of COVID-19, we used four study cohorts to perform a systematic study of cytokine levels in patients with different disease stages. We observed differential cytokine expression between patients with persistent-mild disease and patients with mild-to-severe transformation. For instance, IL-4 and IL-17 levels significantly increased in patients with mild-to-severe transformation, indicating differences within the mild disease group. Subsequently, we analysed the changes in cytokine and chemokine expression in the plasma of patients undergoing two opposing processes: the transition from mild to severe illness and the transition from severe to mild illness. We identified several factors, such as reduced expression of IL-16 and IL-18 during the severe phase of the disease and up-regulated expression of IL-10, IP-10, and SCGF-β during the same period, indicative of the deterioration or improvement of patients’ conditions. These factors obtained from fine-tuned research cohorts could provide auxiliary indications for changes in the condition of COVID-19 patients.

## Introduction

Since the discovery of SARS-CoV-2 at the end of 2019, the novel coronavirus has raged worldwide for nearly 4 years^1–3^. As of March 10, 2023, with the last update from the COVID-19 dashboard by Johns Hopkins University, the disease has resulted in more than 6.8 million deaths worldwide^4^. Notably, clinical events have been observed where the disease rapidly progresses from mild to severe, critical or even fatal stages within a short timeframe, posing a significant threat to the lives and health of COVID-19 patients^5,6^. During the pathogenesis of SARS-CoV-2 infection, virus-induced cell death leads to the release of various pathogen-associated molecular patterns (PAMPs) and damage-associated molecular patterns (DAMPs). These patterns are recognized by pattern recognition receptors on alveolar macrophages and endothelial cells, triggering the increased expression and secretion of inflammatory cytokines and chemokines, such as IL-6, IFN-γ, and MCP1. This process leads to the recruitment and aggregation of immune cells, including macrophages, dendritic cells, and specific T cells, in the lungs to clear virus-infected cells^7–9^. Subsequently, patients transition from having mild symptoms to having severe symptoms, referred to as the hyper-inflammatory stage^10^. During this stage, immune system regulation becomes dysregulated, resulting in excessive production of inflammatory factors, such as IL-6, IL-10, and G-CSF^11–14^. The massive accumulation and infiltration of immune cells in the lungs cause ongoing damage to lung tissues. This pronounced pulmonary inflammation can lead to pulmonary embolism, irreversible lung injury, and severe acute respiratory distress syndrome^15,16^. The dysfunction and failure of organs other than the lungs may also occur, creating a life-threatening situation for the patient^17–20^.

The discovery of accurate and broad-spectrum bio-markers warning of severe COVID-19 is crucial for the timely screening of patients who might develop severe COVID-19 so that clinical interventions may be administered early to minimize the risk of sequelae and mortality. Given the rapid development in LC‒MS/MS, DIA-MS, and other technologies in the biomedical field, along with the secondary utilization and minimally invasive sampling of serum and plasma samples, proteomic, metabolomic, and lipomic analyses of serum and plasma are feasible for studying the pathogenic mechanism, early warning markers, and prognosis of COVID-19. To date, numerous proteins and metabolites related to the development of COVID-19, involving multiple life processes, have been discovered^21–23^. Proteomic studies have revealed changes in pathways, such as inflammation, cardiovascular, coagulation, and cholesterol metabolism pathways, associated with the progression of the disease. Metabolomic analysis has revealed associations between COVID-19 and tryptophan metabolism, energy metabolism, and pyrimidine metabolism. Using multi-omics research, many well-known factors, such as IL-6, CRP, TNFα, and S100A8/A9, have been analysed, and their associations with COVID-19 progression have been revealed^24^.

Multi-omics studies on COVID-19 could also include the analysis of cytokine and chemokine data. However, due to the broad detection capabilities of LC‒MS/MS technology, there is limited focus on the detection of these factors. Cytokines are believed to play a crucial role in the course of SARS-CoV-2 infection and in controlling and resolving viral infection. However, uncontrolled and excessive cytokine production can lead to immune pathogenesis by causing tissue damage throughout the body. Most COVID-19 patients experience mild to moderate symptoms; however, some individuals may experience increased inflammation due to cytokine or chemokine overproduction, known as cytokine release syndrome (CRS). CRS can result in fatal pneumonia and acute respiratory distress syndrome^25,26^. Therefore, a comprehensive understanding of the dysregulation of cytokines and chemokines underlying COVID-19 is crucial for identifying patients at risk of progressing to severe disease, thereby enabling early clinical diagnosis and intervention. Although some previous omic studies have identified the role of cytokines and chemokines in revealing disease progression, these cohort studies usually compare biomarker changes in the same patient during the mild-to-severe period or simply compare the differences between mild and severe patients^27–29^. We posit that “mild is not the same as mild and severe is not the same as severe.” In this study, we systematically established a 4-cohort study comprising 44 patients with different conditions and 84 plasma samples from these patients. By comparing the differences in cytokines and chemokines between patients with persistent mild disease (PM group) and those with mild-to-severe transformation (MS group), patients with persistent severe disease (PS group) and those with severe-to-mild transformation (SM group), and patients with mild-to-severe disease and those with severe-to-mild transformation, we aimed to identify a variety of cytokines and chemokines related to the development of and changes in COVID-19 in these patients. Our data revealed cytokines that can predict the prognosis of COVID-19 during the initial mild or severe phase, and these cytokines have little overlap with the cytokines that change as a patient's disease progresses from mild to severe or from severe to mild disease. These data further increase our understanding of COVID-19 immune dysfunction and reveal cytokines and chemokines that may predict subsequent disease progression in patients with different severities of COVID-19.

## Results

### Study design and patient cohort

**Figure 1:**
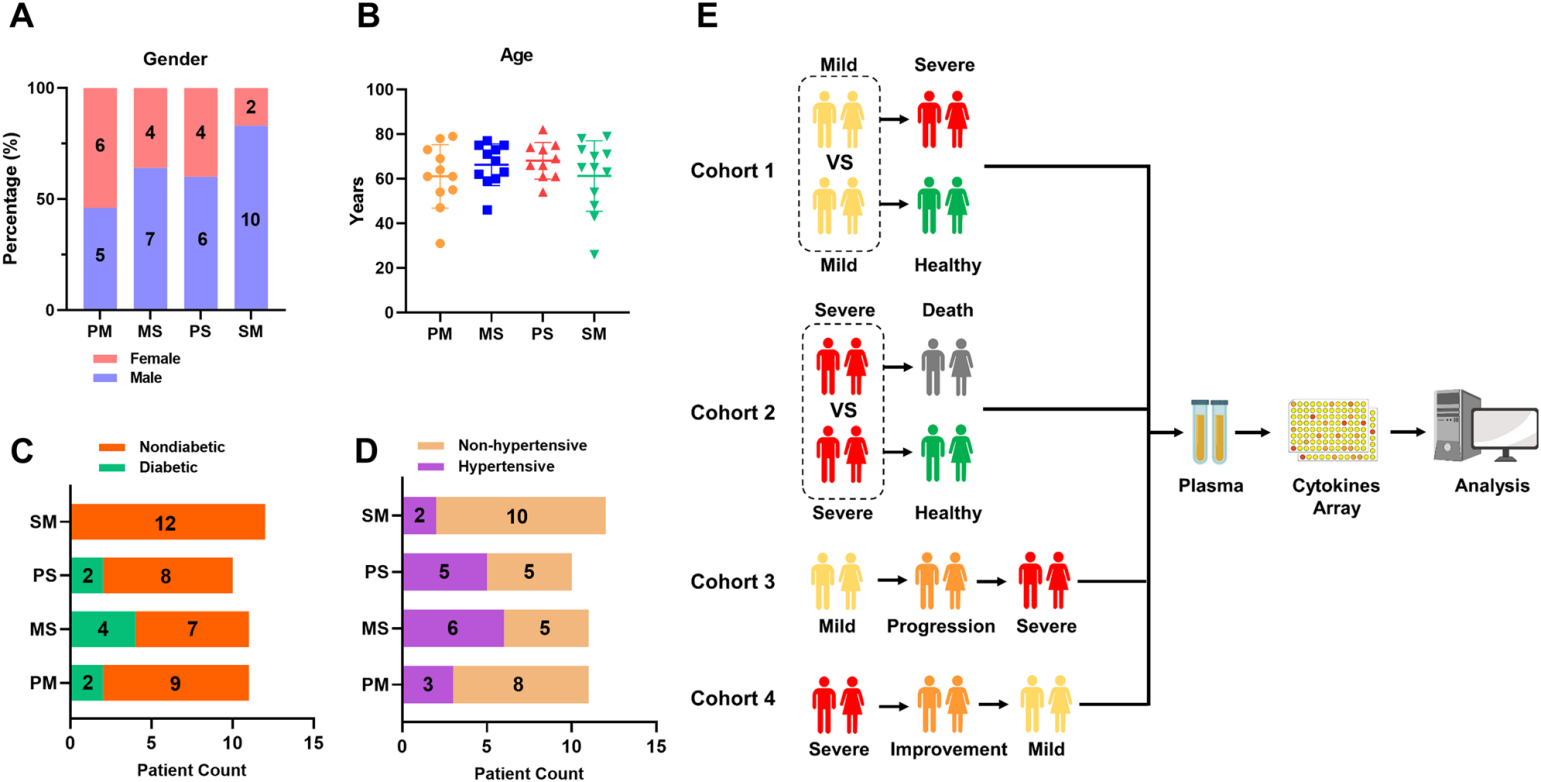
Basic information and configuration of COVID-19 patients in different cohorts, The 44 patients with COVID-19 were divided into four groups: patients with persistent mild disease (PM group), patients with mild-to-severe disease (MS group), patients with persistent severe disease (deceased patients, PS group), and patients with severe-to-mild disease (SM group). (**A**–**D**) The basic information of patients regarding sex, age, hypertension status, and diabetes status was compared between the PM and MS groups and between the PS and SM groups. Fisher’s exact test was used to analyse sex, hypertension status, and diabetes status, while the Wilcoxon rank-sum test was used to analyse age differences among the various groups. No significant differences in clinical information were observed among the groups. *P* > 0.05 indicates no significant difference, and these instances are not marked in the figure. (**E**) Schematic diagram of the four clinical study cohorts: Cohort 1, a comparison of the PM group and MS group; Cohort 2, a comparison of the PS group and SM group; Cohort 3, an analysis of symptoms from mild to severe COVID-19; and Cohort 4, an analysis of symptoms from severe to mild COVID-19.

Forty-four patients who were hospitalized between May 6, 2023, and July 6, 2023, were enrolled in this study. Respiratory tract samples from these participants tested positive for SARS-CoV-2 (Omicron strain) nucleic acid in quantitative real-time polymerase chain reaction (qRT‒PCR) analyses. According to the COVID-19 Infection Prevention and Control Plan (10^th^ Edition), 84 plasma samples from 44 participants were categorized into the following four disease conditions: (1) patients had mild symptoms during sampling, (2) patients had mild-to-severe (progression) or (3) severe-to-mild (improvement) transitional disease during sampling, and (4) patients had severe symptoms during sampling. To comprehensively explore the varying trends in cytokines and chemokines in patients with different disease conditions, four study cohorts were systematically established: Cohort 1 included 11 patients with persistent mild conditions (PM group) and 11 patients with mild-to-severe conditions (MS group). Differences in cytokine levels between patients with mild conditions in the two groups were compared. Cohort 2 included 10 patients with persistent severe conditions (deceased patients; PS group) and 12 patients with severe-to-mild conditions (SM group). Differences in cytokine levels between patients with severe conditions in the two groups were compared. Cohort 3: The varying trends in cytokines in 11 mild-to-severe patients (MS group) were analysed in the mild, progressive, and severe conditions. Cohort 4: The differences in cytokine expression in 12 severe-to-mild patients (SM group) were compared across three stages: severe, improved, and mild disease (Fig. 1E). All plasma samples collected from patients were evaluated using a multiplex system for the simultaneous measurement of the levels of 48 cytokines and chemokines. To explore and compare differences in cytokine levels between patients with initially mild and initially severe conditions in cohort 1 and cohort 2, demographic data for the four patient groups were analysed. There were no significant differences in sex or age between the PM group and the MS group or between the PS group and the SM group (Fig. 1A,B). Similarly, data on diabetes and hypertension for the four patient groups were included in the analysis, and no significant differences were observed in these two underlying diseases among the four groups (Fig. 1C,D).

### Differences in cytokine levels in patients with different clinical outcomes during initial mild or severe conditions

**Figure 2:**
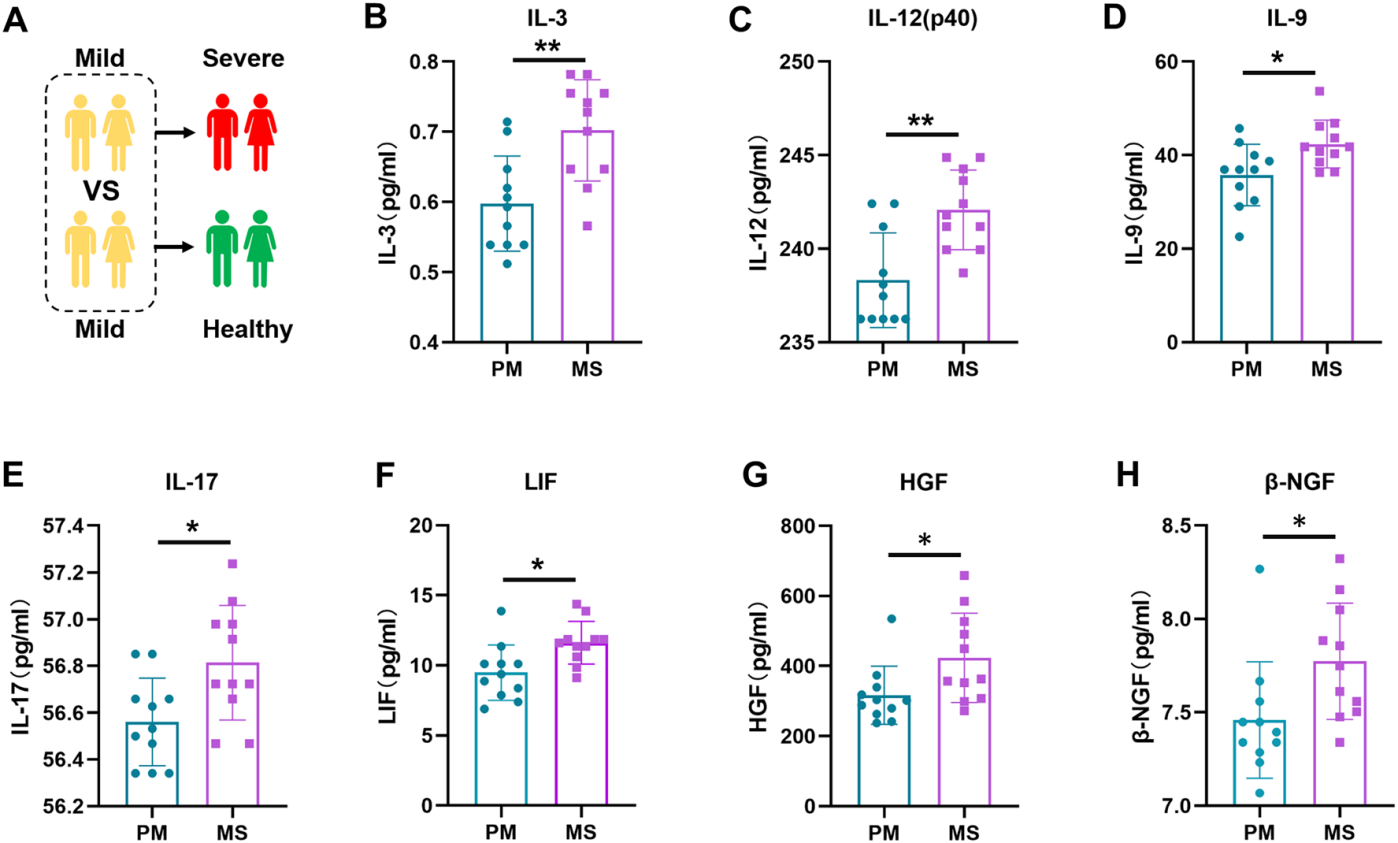
Cytokine and chemokine analysis of patients in the PM and MS groups during the mild stage of COVID-19, (**A**) Schematic diagram illustrating patients in the PM and MS groups. (**B**–**H**) Differences in the expression of IL-3, IL-12 (p40), IL-9, IL-17, LIF, HGF, and β-NGF between patients in the PM and MS groups during the initial mild phase of COVID-19. The* p* value was calculated using unpaired two-sided Student’s *t* tests. *P < 0.05, **P < 0.01.

There are differences in the immune status of patients at the initial mild or severe stage, and these differences determine why some patients are in the mild stage and are ultimately discharged successfully, while some patients progress from having mild to severe disease and even lose their lives. Therefore, cohorts 1 and 2 were established to explore differences in cytokine and chemokine levels in patients with initially mild or severe disease. Initially, we examined the differences in cytokine and chemokine levels between the PM group and MS group during the mild phase (Fig. 2A). The results revealed that, compared with those in the PM group, the levels of seven cytokines were significantly elevated in the MS group during the mild stage, while no significant difference in the down-regulation of the expression of chemokines was found. These seven factors included hepatocyte growth factor (HGF), leukaemia inhibitory factor (LIF), β-nerve growth factor (β-NGF), interleukin 3 (IL-3), interleukin 9 (IL-9), interleukin 12p40 (IL-12p40), and interleukin 17 (IL-17). Figure 2B–H illustrates the differences in the immune status of patients in the PM group and the MS group during the mild phase. The levels of cytokine and chemokines that were not significantly different between the two groups are also illustrated in Figure S1. Additionally, we analysed the levels of immune cells and inflammatory markers in both groups. The results showed that the white blood cell, monocyte, lymphocyte, and platelet counts, indicative of patient immune status, did not differ between the two groups during the initial mild phase. However, c-reactive protein (CRP) and aspartate transaminase (AST) expression levels were significantly up-regulated in the MS group, while alanine aminotransferase (ALT) and creatinine levels did not change (Figure S2).

**Figure 3:**
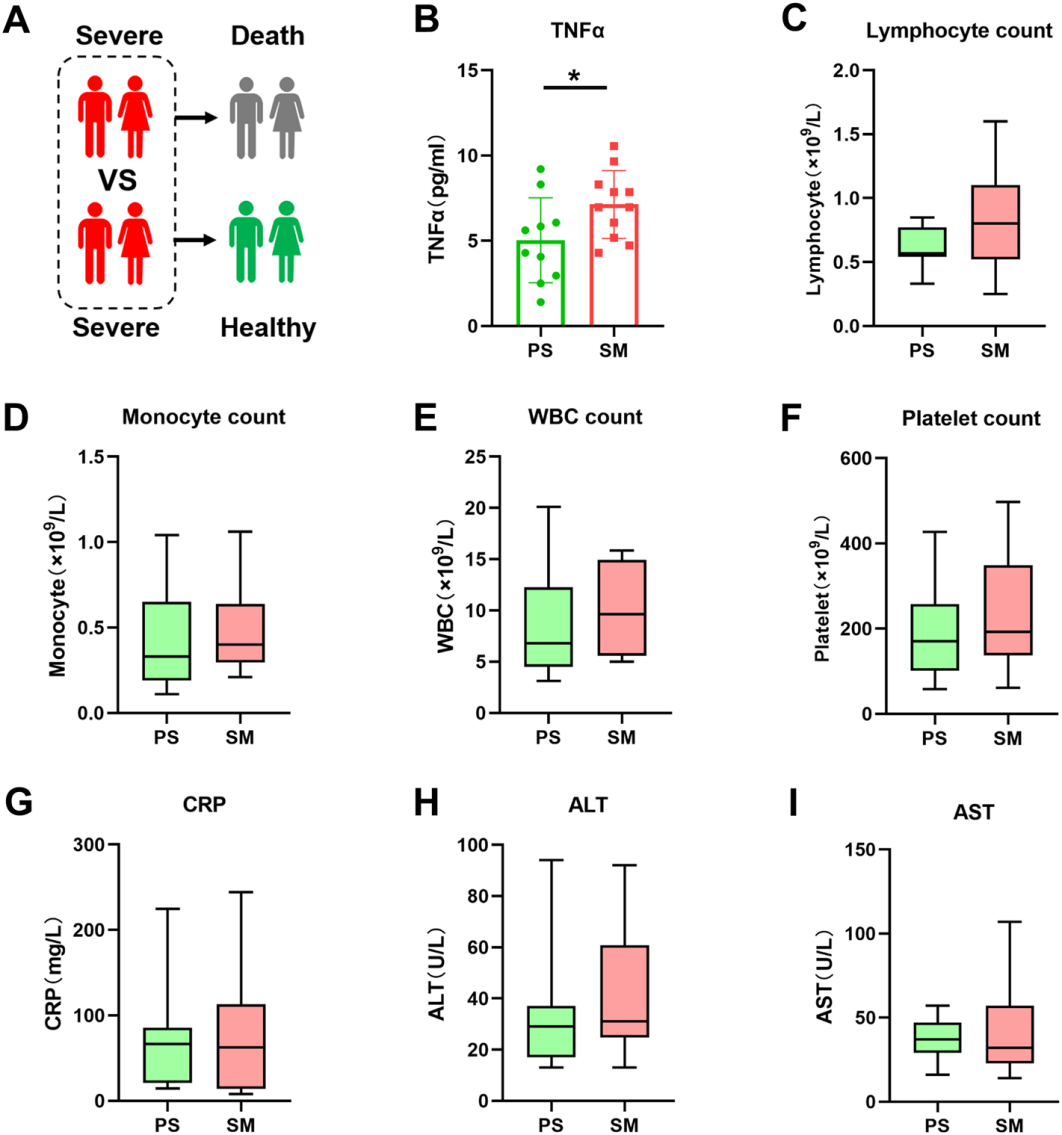
Cytokine and chemokine analysis of patients in the PS and SM groups during the severe stage of COVID-19, (**A**) Schematic diagram illustrating patients in the PS and SM groups. (**B**) Differences in the expression of TNF-α between the PS and SM groups during the initial severe phase of COVID-19. The* p* value was calculated using unpaired two-sided Student’s *t* tests. *P < 0.05. (**C**–**I**) Quantitative analysis of lymphocytes, monocytes, white blood cells, platelets, CRP, ALT, and AST in patients in the PS and SM groups during the severe stage of COVID-19. The* p* value was calculated using unpaired two-sided Student’s *t* tests. *P* > 0.05 indicates no significant difference, and these instances are not marked in the figure.

Subsequently, we analysed the differences in cytokine and chemokine levels between the PS group and the SM group during the initial severe period from another perspective (Fig. 3A). In contrast to the multiple cytokines identified in the analysis of the PM and MS groups, only one cytokine, TNF-α, was differentially expressed between the PS and SM groups (Fig. 3B). As only one cytokine was found to be different in the initial severe period between the two groups of patients, we aimed to further analyse and differentiate the two groups based on other clinical data. We compared the lymphocyte, monocyte, white blood cell, and platelet counts in the PS and SM groups. The clinical characteristics of CRP, ALT, and AST were also included in the comparative analysis. No significant differences were observed in the number of immune cells or clinical indicators (Fig. 3C–I). Additionally, there was no significant difference in the other factors between the PS group and the SM group. The *P* values of the other factors between the two groups were all greater than 0.09, which increased the difficulty of determining the prognosis of COVID-19 based on cytokine levels during the severe period (Figure S3).

### Identification of cytokines for early warning of severe COVID-19

**Figure 4:**
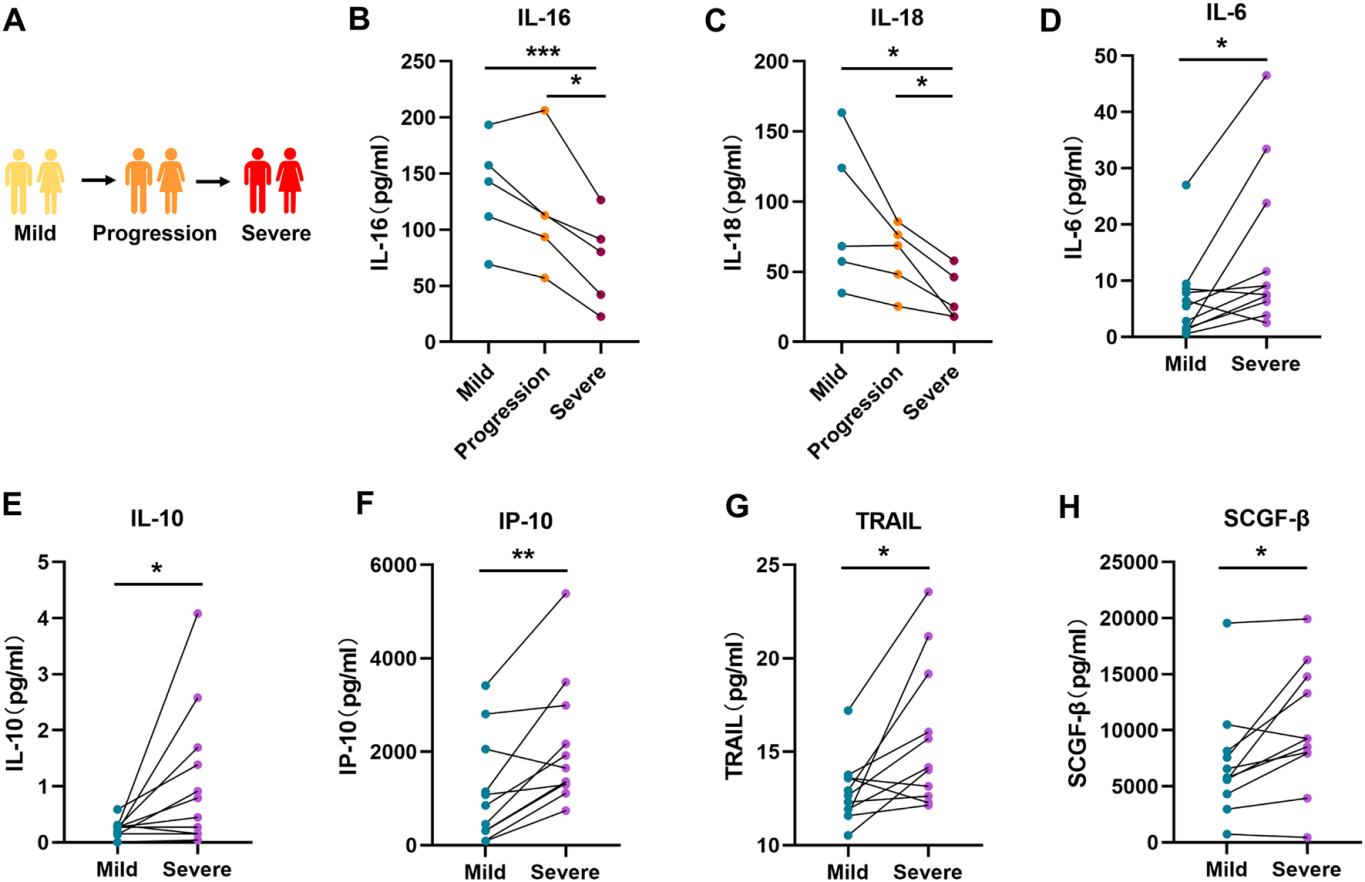
Dynamic trends in cytokine and chemokine levels during disease progression in patients with COVID-19, (A) Schematic diagram illustrating patients undergoing a mild-to-severe transition to COVID-19. (B-H) Plasma levels of IL-16, IL-18, IL-6, IL-10, IP-10, TRAIL, and SCGF-β in patients with mild, progressive, and severe COVID-19. The* p* value was calculated using paired two-sided Student’s *t* tests. *P < 0.05, **P < 0.01.

Beyond examining the differences in immune status among patients with varying disease progression in the initial mild or severe stage, it is crucial to note the gradual changes in cytokine levels during the progression of COVID-19. Cohort 3 consisted of 11 patients with mild-to-severe COVID-19, and the dynamic variations in cytokine and chemokine levels in the plasma of these patients during the mild, progressive, and severe stages were analysed (Fig. 4A). Our results revealed two down-regulated and five up-regulated cytokines/chemokines during the progression from mild to severe COVID-19. The two cytokines that were down-regulated were interleukin 16 (IL-16) and interleukin 18 (IL-18). Significant differences in the two factors were observed in patients transitioning between mild and severe conditions. Additionally, there was a further decrease in expression as patients’ status transitioned from the progressive stage to the severe stage (Fig. 4B,C). Furthermore, the five cytokines and chemokines with up-regulated expression during the progression of COVID-19 were interleukin 6 (IL-6), interleukin 10 (IL-10), interferon gamma-induced protein 10 (IP-10), TNF-related apoptosis-inducing ligand (TRAIL), and stem cell growth factor beta (SCGF-β). Although these cytokines and chemokines did not exhibit significant changes in the progressive stage compared to those in the mild or severe stage, they could clearly distinguish between the mild and severe stages. Therefore, our study revealed that the expression of cytokines, such as IL-6 and IL-10, which have been consistently verified to be involved in the progression of COVID-19 by other studies, is up-regulated as the disease gradually became more severe (Fig. 4D–H). Additionally, we found that the cytokines revealing the transition from mild to severe disease did not overlap with those discovered in patients when comparing the PM and MS groups (Figure S4). These results emphasize the importance of our multi-cohort approach: even patients with mild symptoms may exhibit differences in prognosis that can be detected using real-time analysis of cytokine and chemokine changes.

### Analysis of prognostic cytokines in patients with severe COVID-19

**Figure 5:**
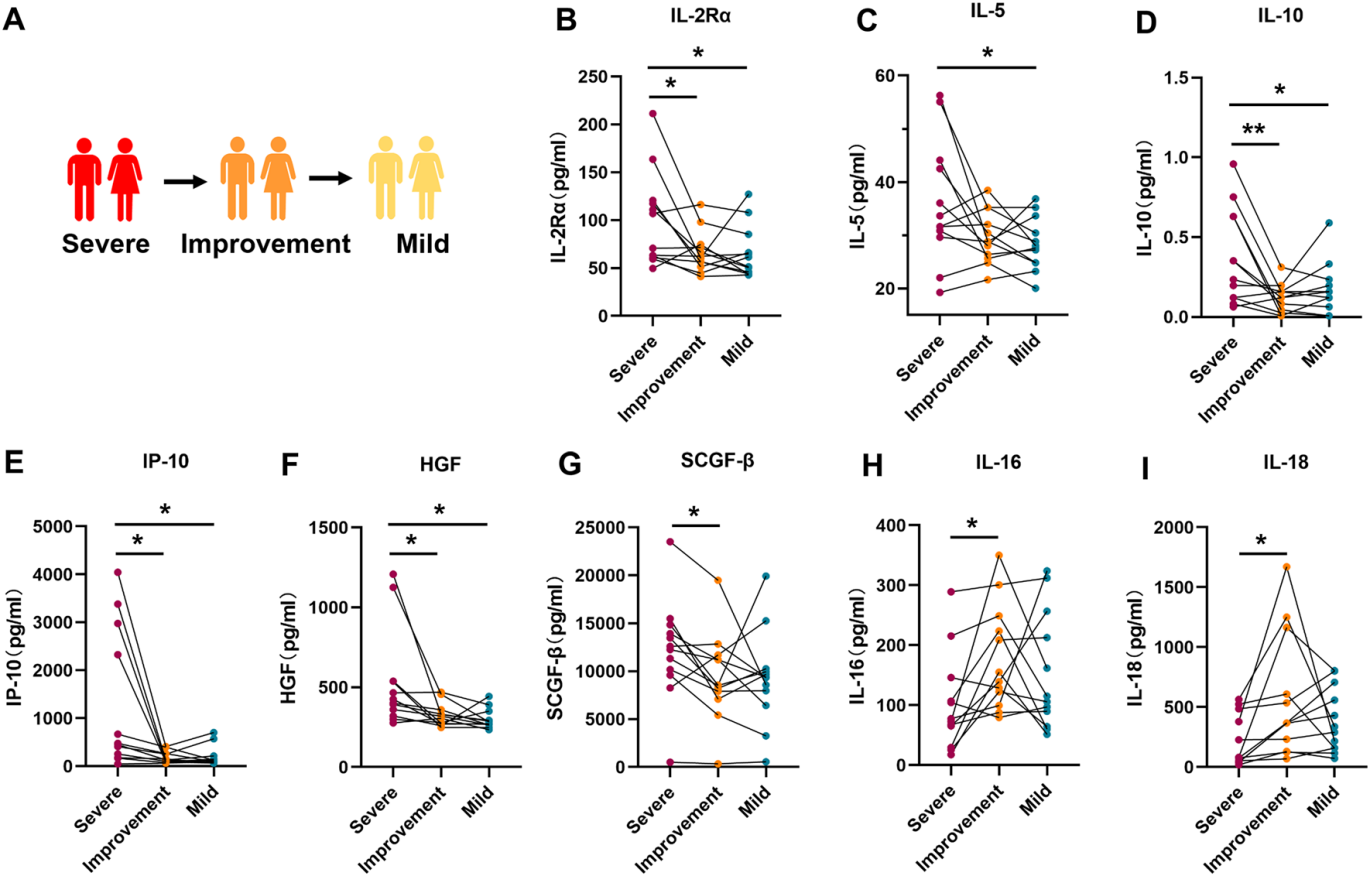
Dynamic trends in cytokine and chemokine levels during COVID-19 improvement. (**A**) Schematic diagram illustrating patients undergoing severe-to-mild transition in COVID-19. (**B**–**I**) Plasma levels of IL-2Rα, IL-5, IL-10, IP-10, HGF, SCGF-β, IL-16, and IL-18 in patients with mild, improved, and severe COVID-19. The* p* value was calculated using paired two-sided Student’s *t* tests. *P < 0.05, **P < 0.01.

The immune status of patients with severe COVID-19 is more intricate than that of patients with mild COVID-19. Patients with severe COVID-19 often experience severe inflammation and receive drug therapy and oxygen support during intensive treatment. Concurrently, underlying disease may worsen during the severe stage, complicating the clinical prognosis of COVID-19 in these patients. Nevertheless, the cytokine storm in patients with severe COVID-19 is a characteristic of severe disease, and subtle changes in cytokines are closely related to disease development, which is highly likely to indicate the prognosis of COVID-19 in these patients. In our study, we established a cohort of 12 patients with severe-to-mild COVID-19, and plasma samples were collected from each patient during the severe, improved, and mild stages (Fig. 5A). Analysis of the dynamic changes in cytokines and chemokines at different disease stages revealed eight process factors related to disease improvement. These cytokines included IL-16 and IL-18, whose expression was up-regulated, and IL-10, IP-10, HGF, SCGF-β, interleukin 2 receptor subunit alpha (IL-2Rα), and IL-16, whose expression was down-regulated as the patients’ conditions improved (Fig. 5B–I). Some of these factors are consistent with the early warning signs of mild to severe disease. For example, IL-16 and IL-18 expression decreased significantly during the transition from mild to severe disease in patients but increased significantly during the transition from severe to mild disease, indicating the consistency of the two cytokines during the disease transition process. Similarly, the expression levels of IL-10, IP-10, and SCGF-β, which were elevated during the progression from mild to severe disease, were significantly decreased when patients’ conditions improved from the severe disease stage. While most of the cytokines and chemokines did not show a statistically significant differential trend during the transition from severe to mild disease (Figure S5), combining these analyses with data on patients transitioning from mild to severe disease revealed multiple common cytokines that varied consistently across both cohorts. The cytokines that showed the same trend in both cohorts included IL-10, IP-10, SCGF-β, IL-16, and IL-18. The expression levels of IL-10, IP-10, and SCGF-β were increased during mild to severe transformation but decreased during severe to mild transformation. Conversely, the expression of IL-16 and IL-18 decreased during the transition from mild to severe disease but increased during the transition from severe to mild disease. Moreover, IL-16 and IL-18 were among the few cytokines whose expression decreased as patients’ disease progressed.

### Correlation between COVID-19-related cytokines and clinical scores

**Figure 6:**
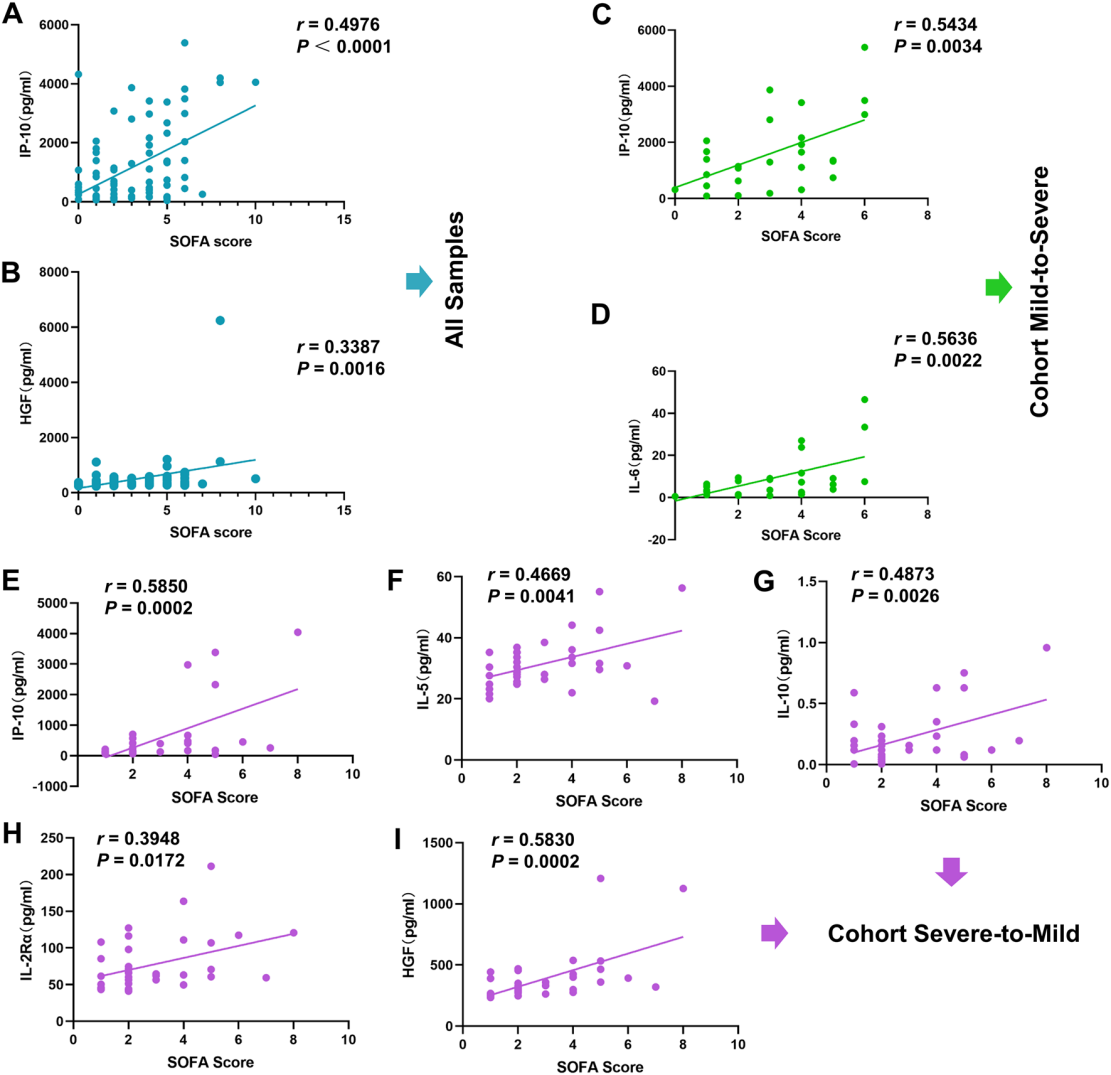
Correlation analysis of cytokines and chemokines with clinical SOFA scores. (**A**–**B**) Correlations between plasma levels of IP-10 and HGF and SOFA scores in all 84 samples from 44 patients. The Pearson correlation coefficient was used to determine the correlation’s *r* value between any two groups. (**C**,**D**) Correlations between plasma levels of IP-10 and IL-6 and SOFA scores in patients who experienced mild-to-severe transition. The data were analysed using the Pearson correlation coefficient. (**E**–**I**) Correlations between plasma levels of IP-10, IL-5, IL-10, IL-2Rα, and HGF and SOFA scores in severe-to-mild COVID-19 patients. The Pearson correlation coefficient was used in the analysis.

In our multi-cohort studies, several indicator cytokines and chemokines that could signify disease progression in COVID-19 patients were identified. To further explore the correlation between these factors and clinical characteristics, as well as to ascertain whether these cytokines can support the clinical diagnosis and treatment of COVID-19, we analysed their correlation with sequential organ failure assessment (SOFA) scores. First, we identified six cytokines and chemokines, including HGF (significant difference in cohort 1 and cohort 4, Fig. 2G and Fig. 5F) and IL-10, IP-10, SCGF-β, IL-16, and IL-18 (significant difference in cohort 3 and cohort 4, Fig. 4 and Fig. 5), that consistently showed variations in COVID-19 in different cohorts. The correlation analysis of SOFA scores for all 84 samples corresponding to the same period of patients and these six cytokines revealed that IP-10 and HGF were correlated with patients’ SOFA scores, with a relatively strong correlation observed for IP-10 (Fig. 6A,B). There was no correlation between the remaining four cytokines and the corresponding SOFA scores in any of the samples (Figure S6A-D). Second, the same six cytokines with consistent variations in COVID-19 in different cohorts and additional cytokines identified in cohorts 3 and 4 were analysed for their correlation with SOFA scores. The levels of two cytokines, IP-10 and IL-6, were moderately correlated with SOFA scores in patients with mild-to-severe disease (Fig. 6C,D). However, the other six cytokines and chemokines, TRAIL, SCGF-β, HGF, IL-10, IL-16, and IL-18, despite having early warning functions in the course of disease development, were not significantly correlated with the clinical SOFA scores (Figure S6E-J). Additionally, IL-2Rα, IL-5, IL-10, HGF, and IP-10 were correlated with SOFA scores in patients with severe-to-mild COVID-19 at different disease stages (Fig. 6E–I). Although IL-16 and IL-18 were the only cytokines that were negatively correlated with SOFA scores in patients at different COVID-19 stages, the results were not statistically significant (Figure S6K and L). The correlation of IP-10 with SOFA scores was validated in all three cohorts. Moreover, IP-10 exhibited different changes and expression patterns in patients with mild-to-severe or severe-to-mild COVID-19. Therefore, IP-10 was identified as a key target in this study and may play a pivotal role in the clinical treatment of COVID-19.

## Discussion

Cytokines and chemokines play multifaceted roles, such as executors and recruiters, in the development of COVID-19. Therefore, the dynamic variations in the expression of these factors at a certain stage often indicate the changing trend of COVID-19. Focusing on the functions of cytokines and chemokines is valuable for providing data for the diagnosis, treatment, and prognosis assessment of COVID-19. We established four cohorts to study cytokines and chemokines that indicate the progression of COVID-19 from two dimensions: patients’ initial immune status and immune status variations during the progression of the disease. In terms of the initial immune status during the same mild phase, the expression of seven cytokines was up-regulated in the MS group compared to the PM group. Among these differentially expressed cytokines, there are no well-known key targets, such as IL-6 and IL-10, involved in the formation of cytokine storms. This finding underscores the significant importance of our study in elucidating the differences in immune status among patients with various COVID-19 outcomes in the initial mild stage. Due to the disparities between cross-sectional and longitudinal studies, the expression level and change trend of the same cytokine may be completely different during the initial immune state and disease development. For instance, in our study, we observed that high expression of IL-3 in patients at the mild stage was associated with severe COVID-19, while other studies have reported contrasting findings, indicating that low IL-3 expression is associated with increased severity, viral load, and mortality from COVID-19^30^. Another study revealed that the expression of IL-3 in healthy individuals was greater than that in patients with COVID-19, but survivors of severe COVID-19 exhibited increased serum concentrations of IL-3 compared to those in non-survivors^31^. In our study, seven cytokines and chemokines, including IL-12p40, were expressed at higher levels in the MS group than in the PS group. One study confirmed that IL-12p40 expression was decreased in patients with severe COVID-19 compared to patients with moderate disease^32^. In contrast, another study revealed that the viral Ct value was negatively correlated with the IL-12p40 level in patients, and the plasma IL-12p40 level was greater in patients with severe COVID-19 than in patients with mild disease^33^. The expression levels of five cytokines, IL-9, IL-17, LIF, HGF, and β-NGF, were significantly greater in the MS group than in the PM group, consistent with findings from previous studies. IL-9, a common γ chain family cytokine primarily produced by Th9 cells, contributes to and exacerbates the viral spread and airway inflammation caused by SARS-CoV-2. Exogenous IL-9 increases airway inflammation in Foxo1-deficient mice, while IL-9 blockade suppresses the progression of SARS-CoV-2 infection^34,35^. One study suggested that IL-17 levels in intensive care patients are greater than those in non-intensive care patients, and acute lung injury in patients who died from the influenza virus was associated with an increase in airborne neutrophils promoted by IL-17^36,37^. LIF is recognized as essential for countering the cytokine storm in the lungs during viral pneumonia, thereby strengthening lung resistance to COVID-19 and reducing the risk of severe disease^38,39^. Therefore, the increased expression of many cytokines, such as LIF, does not cause inflammation but rather results from the suppression of the cytokine storm. Studies on HGF have shown that HGF is associated with severe COVID-19 and can predict the severity and mortality of patients with COVID-19^40,41^. Similarly, β-NGF is expressed at higher levels in intensive care unit (ICU) patients than in non-ICU patients^42^. In general, although symptom changes were not observed in patients in the MS group in the mild stage, a variety of cytokines and chemokines in the body were activated. This prompted the establishment of a comparative cohort study between the PM and MS groups.

Furthermore, the results of another cross-sectional study were “not satisfactory.” When comparing the immune status of patients with COVID-19 in the PS group and SM group at the initial stage of severe disease, the only discernible difference between the two groups was in the expression of TNF-α in plasma. The expression of TNF-α, a pro-inflammatory cytokine that is commonly up-regulated in acute lung injury, can trigger CRS and promote the interaction of SARS-CoV-2 with angiotensin-converting enzyme 2 (ACE2)^43^. Longitudinal serum cytokine analysis of 207 COVID-19 patients revealed that in early inflammatory responses, IL-6, TNF-α, IL-10, and IL-1β expression increased in those with severe disease^44^. Another study demonstrated that COVID-19 patients experienced significant remission after treatment with TNF-α inhibitors, such as infliximab^45^. Our results showed that TNF-α is the only factor among the 48 cytokines and chemokines that can distinguish the initial immune status between the PS and SM groups, and the *P* values of differences in the other factors between the two groups were not even lower than 0.09. Additionally, there were no significant differences in the counts of multiple immune cells or in CRP, ALT, or AST levels between the two groups of patients with severe COVID-19. Therefore, it is difficult to determine the prognosis of severe disease based on cytokines or blood indicators, but TNF-α has potential for prognostication in severe patients.

In the longitudinal analysis of patients’ disease development, we found that the levels of five cytokines and chemokines, IL-10, IP-10, SCGF-β, IL-16, and IL-18, significantly changed during the progression from mild to severe and from severe to mild COVID-19. IL-10 is a pivotal cytokine related to the regulation of inflammation in the development of COVID-19. Numerous studies have demonstrated that IL-10 and IL-6 are important factors for predicting the exacerbation of COVID-19 and are significantly positively correlated with the severity of disease and the mortality rate of patients with COVID-19^12,46,47^. However, although we found that IL-6 expression increased during the course of mild-to-severe COVID-19 transformation, there was no significant change in severe-to-mild transformation. Thus, confirmation of this finding in future studies with large participant samples is warranted. The expression of the chemokine IP-10 was a noteworthy finding in this study. Although previous studies have linked IP-10 to the development of severe COVID-19, its role in early warning of disease development has not been fully explored, in contrast to cytokines such as IL-6 and TNF-α, which have been widely studied^48,49^. In our study, IP-10 exhibited a similar trend of differential expression changes in both the mild-to-severe and severe-to-mild longitudinal study cohorts, with relatively high expression in patients with severe disease. Additionally, in subsequent studies on the correlation between cytokine levels and clinical SOFA scores, IP-10 was the only factor that was positively correlated with SOFA scores across all samples, the mild-to-severe cohort, and the severe-to-mild cohort (Fig. 6A,E,C). Additionally, previous studies have shown that SCGF-β is highly expressed in patients with severe COVID-19, and SCGF-β is positively correlated with SOFA scores. Although our study found that the expression of SCGF-β increased with the deterioration of the COVID-19 disease and decreased with the improvement of the disease, we could not determine whether there was a significant positive correlation between SCGF-β and SOFA scores (*r* = 0.2935, *P* = 0.1373; Figure S6J). In addition to cytokines whose expression increased with the progression of COVID-19, IL-16 and IL-18 were the only two cytokines whose expression decreased significantly with the worsening of the disease. The findings of previous studies on IL-16, in which IL-16 expression was significantly lower in the moderate COVID-19 group than in the acute respiratory distress syndrome (ARDS) and sepsis groups^50^, align with our results. Conversely, previous studies on IL-18 present findings that conflict with our results. One study showed that serum IL-18 expression was markedly greater in patients with COVID-19 than in healthy individuals, with the highest levels observed in the severe pneumonia group^51^. Another study reported that ICU patients with severe COVID-19 display higher levels of IL-18 than patients with moderate disease^52^. However, while previous studies compared two groups of people with different COVID-19 conditions, our study dynamically analysed the change from mild to severe disease and from severe to mild disease in the same patient population. In addition to the aforementioned factors, HGF was also a recurring factor in different cohort analyses, displaying both high expression in the initial mild phase of the MS group and decreased expression as patients’ conditions gradually improved (Fig. 2G and Fig. 5F).

In conclusion, to comprehensively investigate the roles of cytokines and chemokines in the development and prognosis of COVID-19, we meticulously designed four study cohorts consisting of two cross-sectional and two longitudinal study cohorts. Six cytokines and chemokines, IL-10, IP-10, HGF, SCGF-β, IL-16, and IL-18, were identified for analysing the progression patterns of COVID-19, especially IP-10, which was closely correlated with SOFA scores. IP-10 emerged as a potential target chemokine for predicting the prognosis of COVID-19 in various stages. This study provides a new cohort study strategy for different clinical scenarios of COVID-19 and yielded new data variations in cytokines and chemokines. These insights will contribute to advancing the field of research on COVID-19 biomarkers.

### Limitations of the study

Due to the small number of patients with mild COVID-19 hospitalized, especially those with mild to severe cases, only 44 hospitalized patients were included in this study. The small sample size may have resulted in the data not being representative of a broader demographic or geographic area. In addition, this study focused on the Omicron variant of SARS-CoV-2, and the clinical characteristics and induced immune response in patients infected with the Omicron variant may be different from those of patients infected with other SARS-CoV-2 strains. In addition, although we collected plasma samples from patients at different time points, the expression of some cytokines and chemokines may not have been analysed at the optimal times. In the future, it will be necessary to collect clinical samples at more frequent time points to support our conclusions.

## Materials and Methods

### Human subjects

Forty-four hospitalized patients who were diagnosed with COVID-19 between 6/5/2023 and 6/7/2023 were recruited. Among the 44 patients, 40 underwent testing for at least two other respiratory pathogens, but no pathogens other than SARS-CoV-2 were detected. All 84 plasma samples were taken from 44 patients at different stages of the disease. These 44 patients were categorized into four groups (the PM, MS, PS, and SM groups) based on the disease stage, and the demographic and clinical characteristics of each group of patients were collected and analysed (Table S1). In general, 84 samples from 44 patients were classified according to three different disease stages based on the COVID-19 Infection Prevention and Control Plan (10th Edition) issued by China, namely, the mild stage, the progressive/improved (moderate) stage, and the severe stage of COVID-19. This study conformed to the 1975 Declaration of Helsinki guidelines and was approved by the Ethics Committee of Wuhan Infectious Disease Hospital (KY-2023–21.01). Written informed consent was obtained from all involved patients.

### Inclusion criteria and exclusion criteria

Patient characteristics and clinical information were collected. The inclusion criteria were SARS-CoV-2 infection and patient age of 18–82 years. Eighty-four plasma samples from 44 patients were analysed and classified into three different disease stages based on the collected information. (1) Mild stage: Patients with dry throat, sore throat, cough, fever, or other symptoms. (2) Progression/improvement (moderate) stage: Patients with fever sustained for > 3 days and/or cough, shortness of breath, etc., but with a respiratory rate (RR) of < 30 breaths/min, an oxygen saturation of the finger when breathing air at rest was > 93%, and imaging showing the characteristic manifestations of COVID-19 pneumonia. (3) Severe stage: Patients with any of the following criteria that could not be explained by anything other than COVID-19: 1. Shortness of breath, with an RR ≥ 30 breaths/min; 2. At rest, an oxygen saturation ≤ 93% when inhaling air; and 3. An arterial partial oxygen pressure (PaO2)/oxygen absorption concentration (FiO2) ≤ 300mmHg (1 mmHg = 0.133 kPa). In the pursuit of ensuring the integrity and validity of our study while also mitigating potential confounding variables, we established explicit exclusion criteria. These criteria are as follows: (1) pregnancy or lactation; (2) the presence of any other acute infectious disease; and (3) the presence of various severe disease such as chronic lung disease, coronary heart disease, heart failure, kidney failure or liver disease. Moreover, considering the disease development of the patients, 84 samples were divided into the PM, MS, PS, and SM groups and divided into four study cohorts.

### Collection of plasma samples

All 44 patients underwent the first sampling on the day of admission, and the interval between symptom onset and hospital admission was recorded, along with the patients' chief complaints. According to the analysis, there was no significant difference in the interval between different groups (Figure S7A). In addition, we separately plotted graphs showing the intervals from symptom onset to different sampling time points for patients in the PM, MS, PS, and SM groups (Figure S7B-D). Whole blood samples were collected using anticoagulant medical collection tubes (Cat #E5460, Aosite, Inc.), and the samples were centrifuged at 3000 r/min for 3 min at room temperature. After centrifugation, the whole blood sample was divided into upper plasma, middle white membrane and lower red blood cells. The upper plasma was collected and divided into a “three-code in one” frozen storage tube (Cat #88–9150, Aosite, Inc.) customized by the hospital biobank and stored at -80 degrees Celsius until use.

### Measurement of cytokine and chemokine levels

The levels of plasma cytokines were analysed using Bio-Plex (Bio-Rad Laboratories, Inc.) multiplex magnetic bead-based antibody detection kits following the manufacturer’s instructions. Bio-Plex Pro Human Cytokine 48-Plex Screening Panels (Cat #12007283) were used for the analysis of 48 cytokines and chemokines according to the protocols provided by the manufacturer. The core technology of this detection method involves labelling tiny particles, also known as microspheres or beads, with different fluorescent colours. Subsequently, protein or oligonucleotide probes targeting different analytes are covalently attached to microspheres of different colours. The microspheres have a diameter of 5.6 µm and are manufactured by strictly mixing two different fluorescent dyes in specific proportions, allowing the classification of spherical matrices into 100 types based on varying ratios. Each type is labelled with a different probe molecule, enabling the simultaneous measurement of the levels of up to 100 different target molecules in a single sample. The 48 plasma cytokines and chemokines evaluated included FGF basic, Eotaxin, G-CSF, GM-CSF, IFN-γ, IL-1β, IL-1Ra, IL-1α, IL-2Rα, IL-3, IL-12 (p40), IL-16, IL-2, IL-4, IL-5, IL-6, IL-7, IL-8, IL-9, GRO-α, HGF, IFN-α2, LIF, MCP-3, IL-10, IL-12 (p70), IL-13, IL-15, IL-17A, IP-10, MCP-1 (MCAF), MIG, β-NGF, SCF, SCGF-β, SDF-1α, MIP-1α, MIP-1β, PDGF-BB, RANTES, TNF-α, VEGF, CTACK, MIF, TRAIL, IL-18, M-CSF, and TNF-β. The detection ranges for the aforementioned 48 cytokines and chemokines are shown in Table S2. All plasma samples were inactivated at 56 °C for 30 min before the assay.

### Calculation of SOFA scores

The SOFA score is calculated from six different components, each of which reflects the status of an organ system, including respiratory function, cardiovascular integrity, liver function, coagulation, renal function and neurological status. The score can range from 0 (best) to 24 (worst).

### Statistical analysis

Unpaired two-sided Student’s t tests were used to compare the differences between two unpaired groups. Paired two-sided Student’s t tests were used to compare the significance of the paired samples. Patient sex, hypertension status and diabetes status were analysed by Fisher's exact test, and the Wilcoxon rank sum test was used to evaluate the differences in age among patients in various groups. A P value less than 0.05 was considered significant (*: *P* < 0.05; **: *P* < 0.01; ***: *P* < 0.001). The Pearson correlation coefficient (r) and the probability *P* value were calculated using GraphPad Prism, version 8.

### Supplementary Information

Supplementary Information.

## Data Availability

The datasets used and analysed during the current study are available from the corresponding author on reasonable request.
