# Supplementary material for: Multi-cohort study on cytokine and chemokine profiles in the progression of COVID-19
Source: Sci Rep. 2024 May 6;14:10324. doi: 10.1038/s41598-024-61133-z (PMC11074324; doi:10.1038/s41598-024-61133-z)
Supplement: Supplementary file 1 — Supplementary Information. [file 41598_2024_61133_MOESM1_ESM.docx]

**Supplemental Figures and Tables**

**
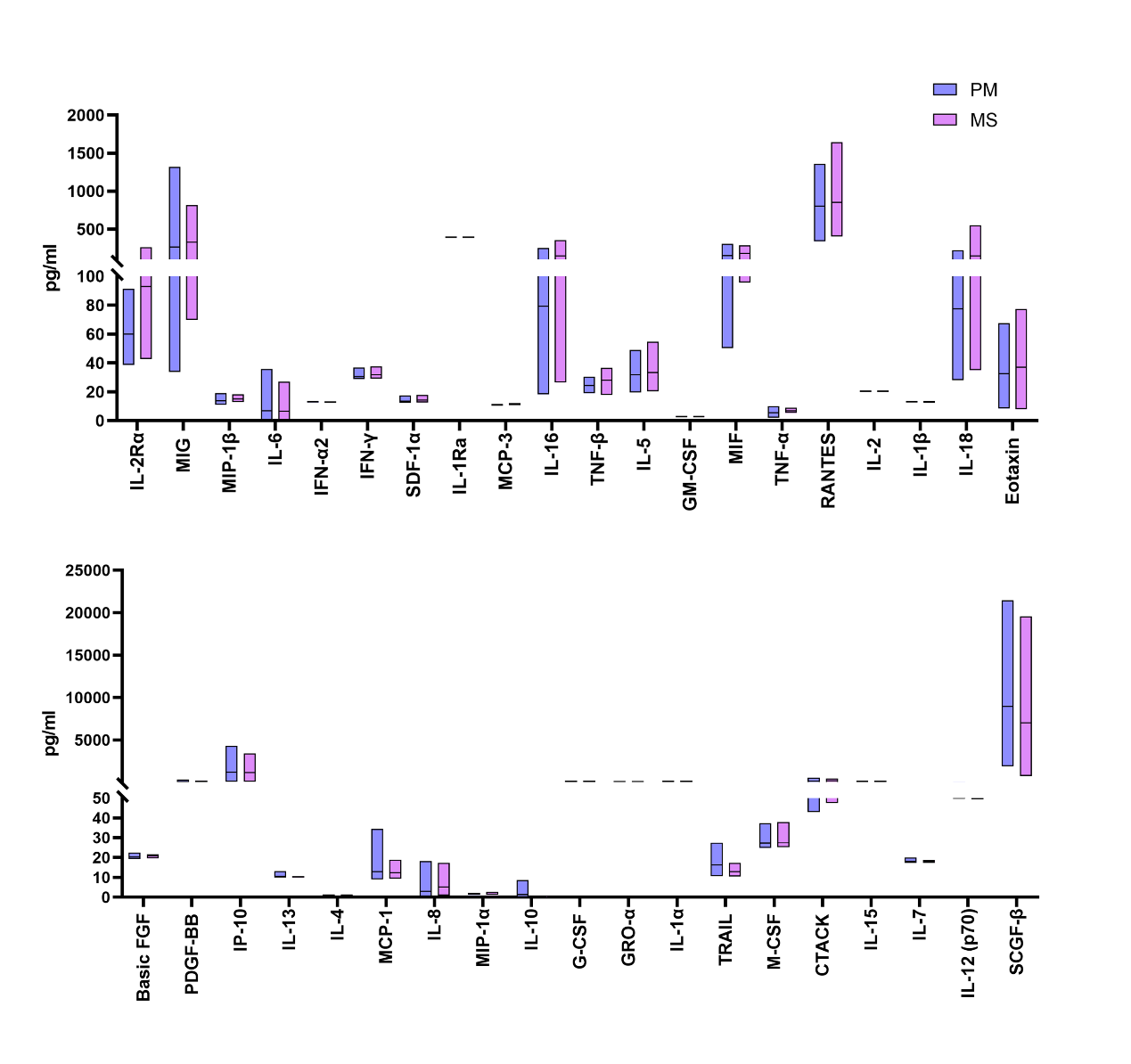
**

**Figure S1. Plasma levels of cytokines and chemokines without significant differences between the PM and MS groups.**

Results of the comparative analysis of the expression levels of 39 cytokines and chemokines between the PM and MS groups of patients with COVID-19. The *p* value was calculated using unpaired two-sided Student’s *t* tests, and the values for all factors in the analysis of significant differences between the two groups were greater than 0.05.


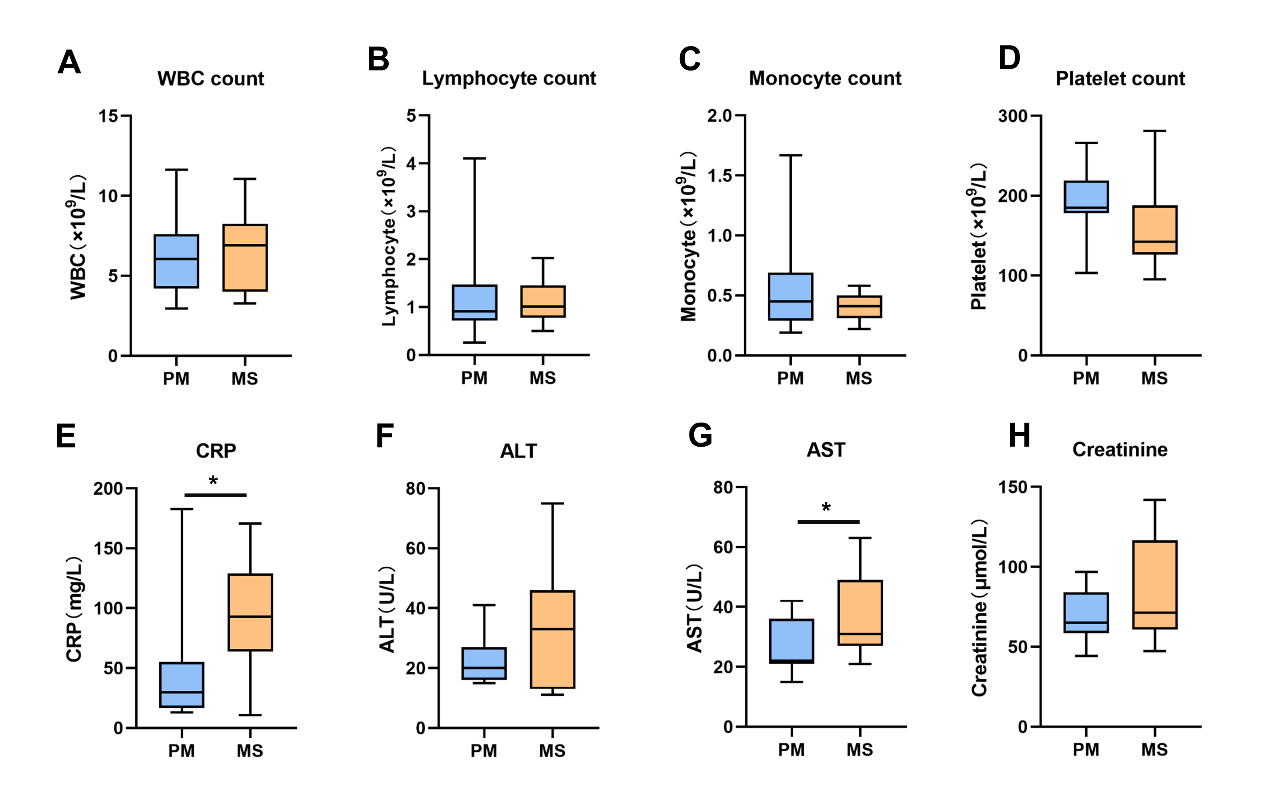


**Figure S2. Results of the comparative analysis of blood cell counts and levels of various blood parameters between the PM and MS groups**

(A-D) Results of the comparison of white blood cell, lymphocyte, monocyte, and platelet counts between the PM and MS groups. The *p* value was calculated using unpaired two-sided Student’s *t* tests. *P* > 0.05 indicates no significant difference, which is not marked in the figure. (E-H) Results of the comparison of blood levels of CRP, ALT, AST, and creatinine between the PM and MS groups. The *p* value was calculated using unpaired two-sided Student’s *t* tests. **P* < 0.05. *P* > 0.05 indicated no significant difference.


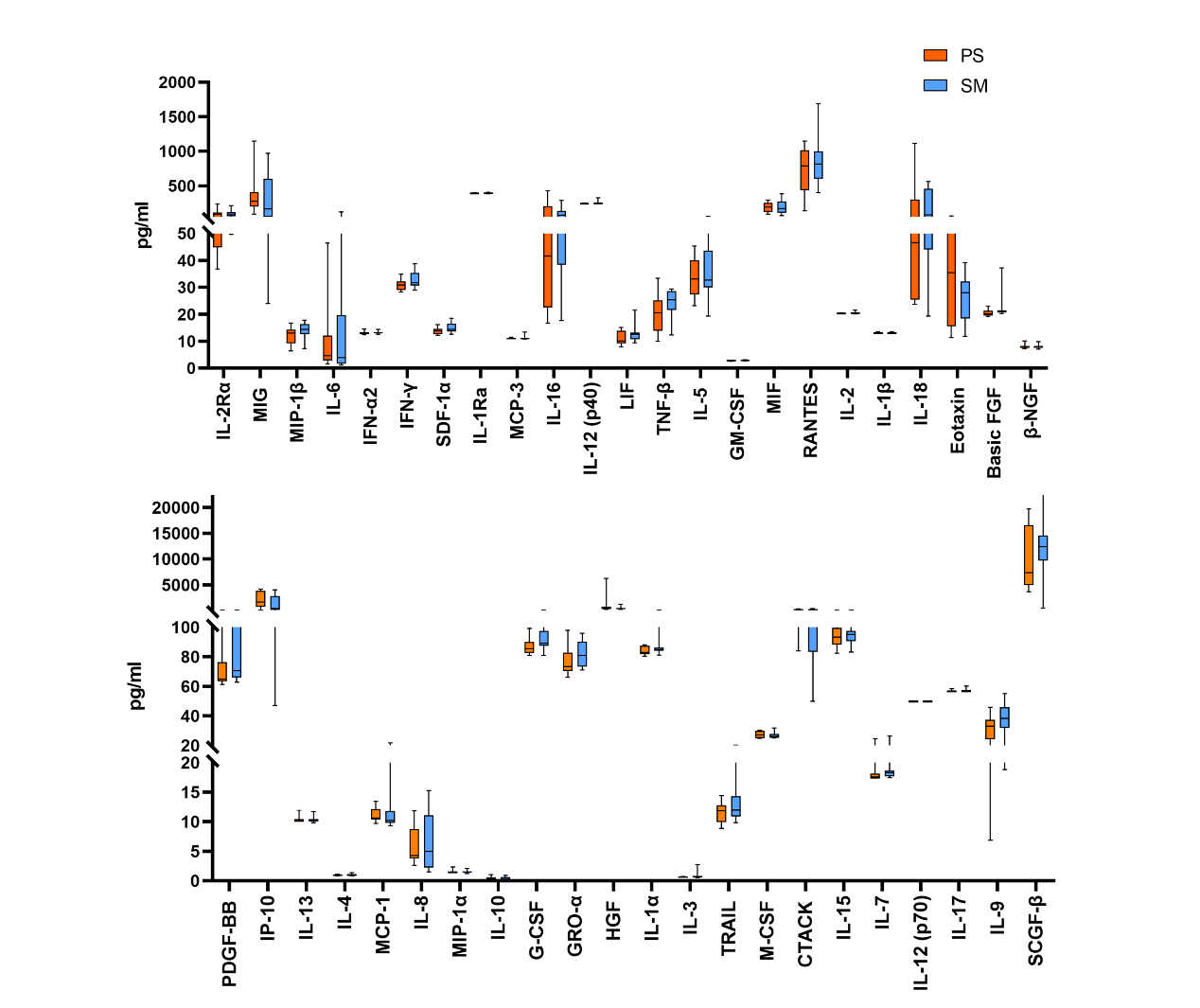


**Figure S3. Cytokines and chemokines without significant differences in plasma levels between the PS and SM groups.**

Results of the comparative analysis of the expression levels of 45 cytokines and chemokines between the PS and SM groups. *P* > 0.05 indicates no significant difference, which is not marked in the figure.


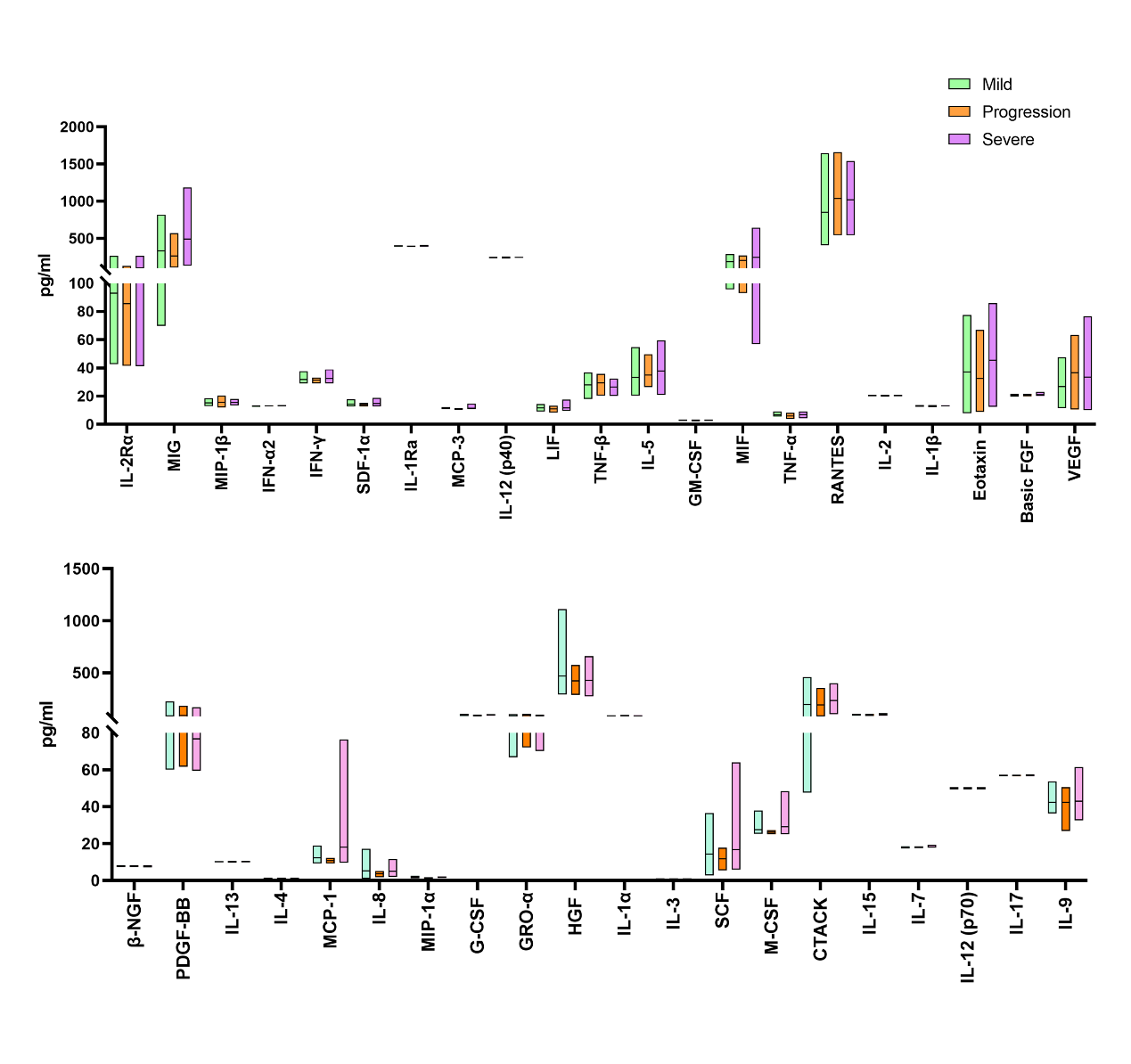


**Figure S4. Cytokines and chemokines without significant differences during the transition of COVID-19 from mild to severe disease**

The dynamic changes in 41 cytokines and chemokines during the transition from mild to severe COVID-19 were analysed. The *p* values were calculated using paired two-sided Student’s *t* tests. *P* > 0.05 indicated no significant difference.


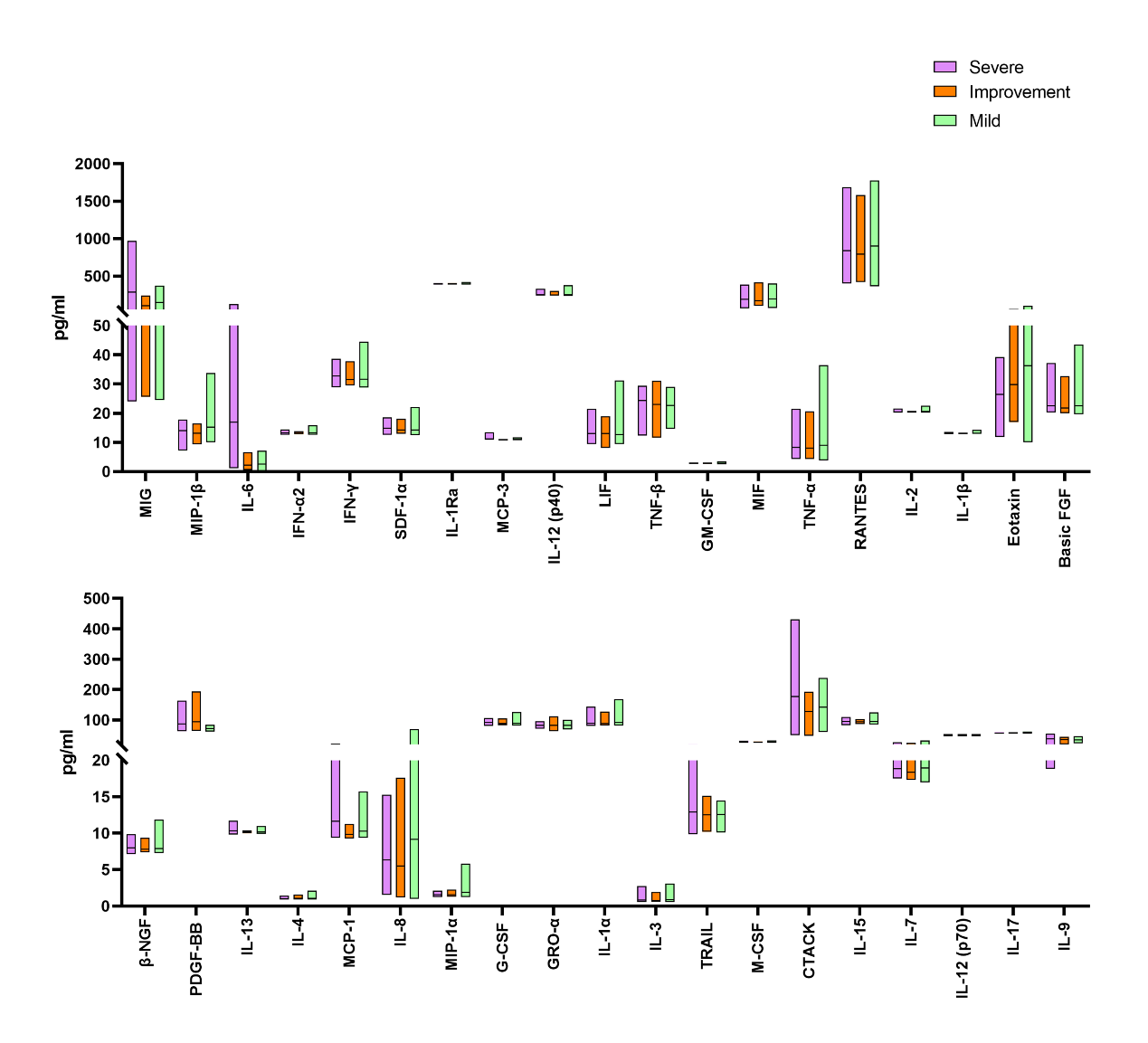


**Figure S5. Cytokines and chemokines without significant changes during the transition from severe to mild COVID-19.**

The dynamic changes in 38 cytokines and chemokines during the transition from severe to improved and from improved to mild COVID-19 were analysed. The *p* value was calculated using paired two-sided Student’s *t* tests. *P* > 0.05 indicated no significant difference.


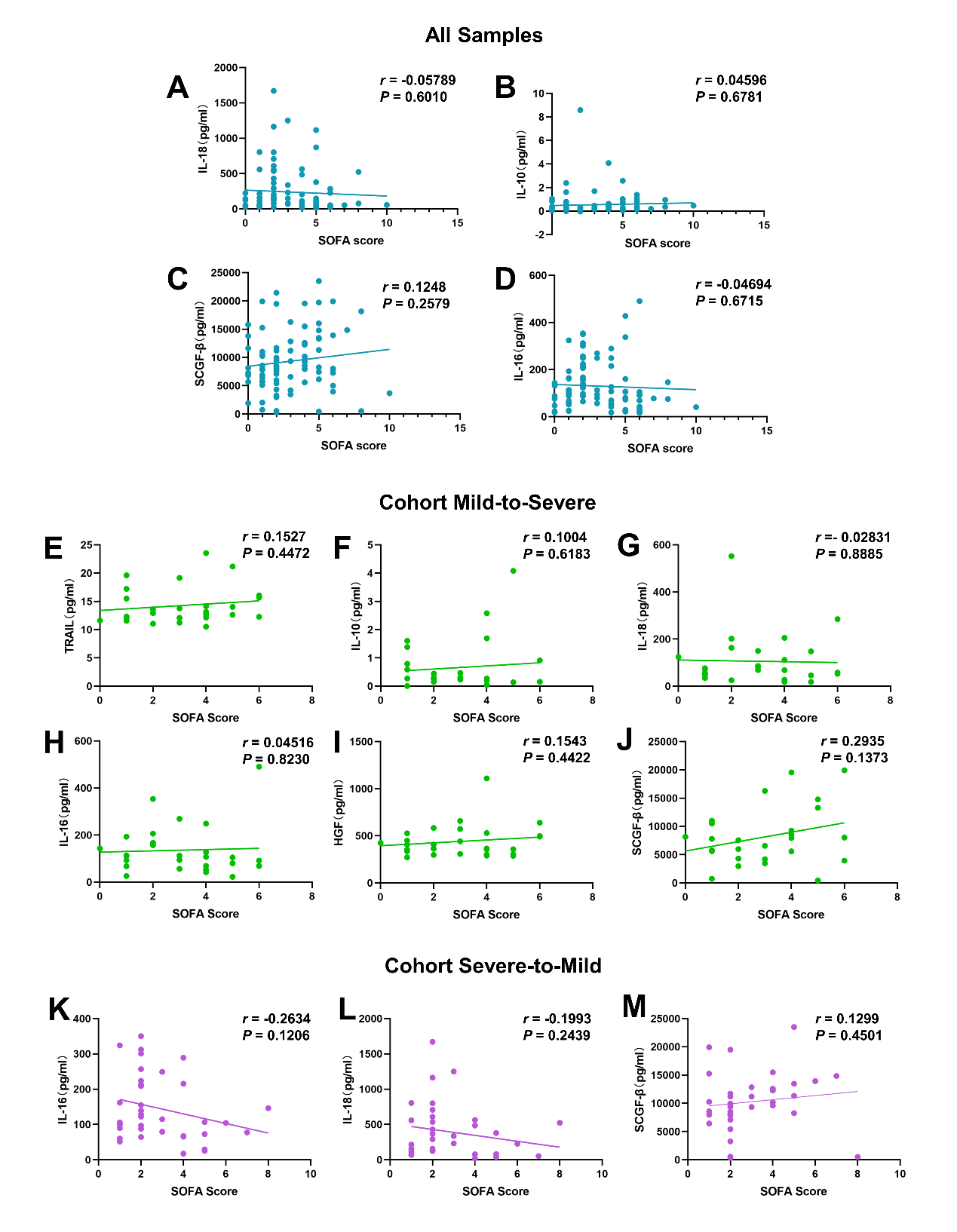


**Figure S6. Cytokines and chemokines with no significant correlation with clinical SOFA scores.**

Correlations between plasma levels of cytokines and chemokines and SOFA scores in all samples (A-D), samples from the mild-to-severe cohort (E-J), and samples from the severe-to-mild cohort (K-M).


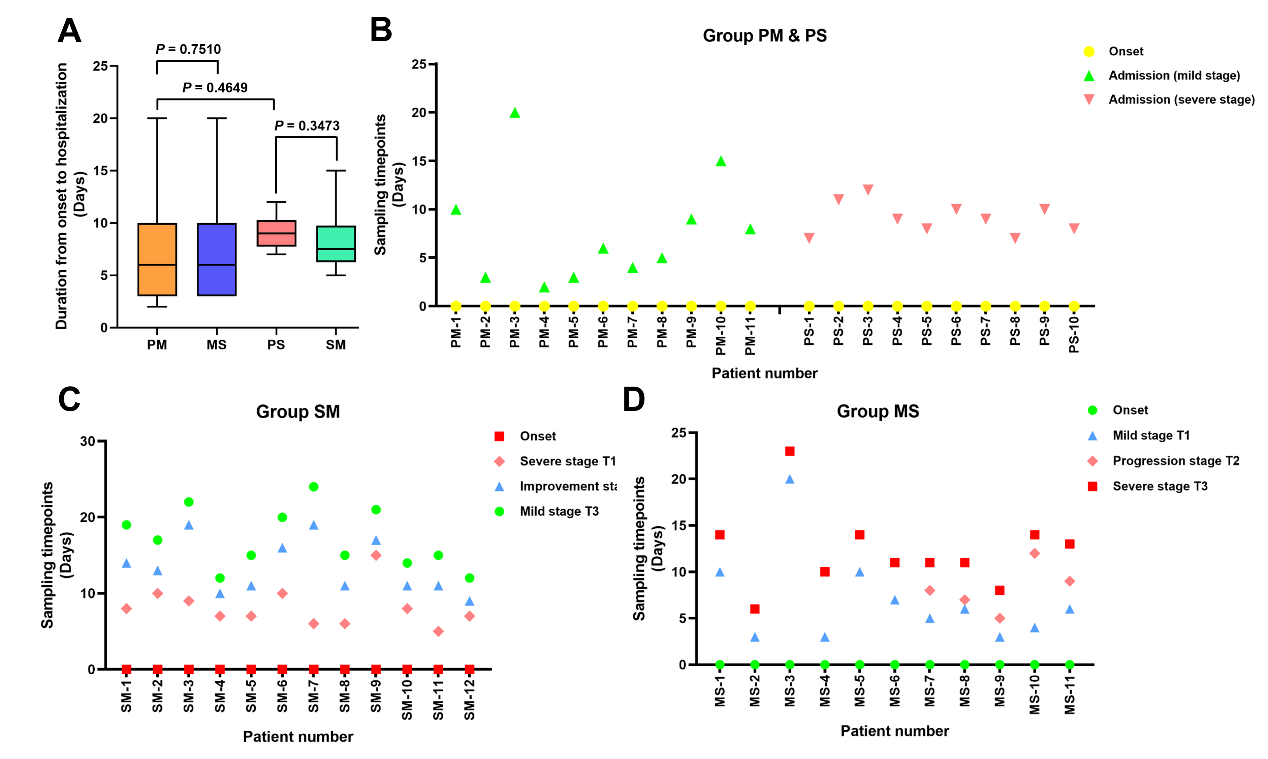


**Figure S7. Schematic diagrams of the time intervals from symptom onset to different sampling time points for the four groups of patients.**

(A) The time intervals from symptom onset to hospital admission for the four groups of patients. The *p* value was calculated using unpaired two-sided Student’s *t* tests. *P* > 0.05 indicated no significant difference. (B-D) Schematic diagrams of all sampling time points for the 84 samples from the four groups of 44 patients. Samples from the PM group and PS group were collected only when the patients were hospitalized (B). The sampling time points for the SM group patients included severe stage T1 (admission), improvement stage T2 and mild stage T3 (C). In theory, the sampling time points for the MS patients included mild stage T1 (admission), progression stage T2 and severe stage T3. However, out of the eleven MS patients, clinical samples were not obtained during the progression period of COVID-19 for six patients with rapid worsening of the disease (D).

Table S1 Demographics and baseline characteristics of COVID-19 patients

|  |  | **Mild illness on admission** | |  | **Severe illness on admission** | |  |
| --- | --- | --- | --- | --- | --- | --- | --- |
| **Variables** | **class** | **Group PM** | **Group MS** | ***p*-value** | **Group PS** | **Group SM** | ***p*-value** |
| n |  | 11 | 11 |  | 10 | 12 |  |
| Age |  | 61.0 [54.5, 71.0] | 68.0 [61.0, 74.0] | 0.3234 | 67.5 [62.2, 73.8] | 65.0 [52.5, 71.5] | 0.3727 |
| Sex (%) | 0  1 | 5 (45.45)  6 (54.55) | 7 (63.64)  4 (36.36) | 0.6699 | 6 (60.00)  4 (40.00) | 10 (83.33)  2 (16.67) | 0.3476 |
| WBC (×10^9^/L) |  | 6.1 [4.4, 7.1] | 6.9 [4.6, 7.9] | 0.8572 | 8.0 [5.3, 11.7] | 9.6 [6.0, 14.9] | 0.6112 |
| Lymphocytes(×10^9^/L) |  | 0.9 [0.8, 1.3] | 1.0 [0.9, 1.4] | 0.6935 | 0.6 [0.5, 0.6] | 0.8 [0.6, 1.0] | 0.0927 |
| Monocytes(×10^9^/L) |  | 0.4 [0.3, 0.7] | 0.4 [0.3, 0.5] | 0.6217 | 0.4 [0.2, 0.6] | 0.4 [0.3, 0.6] | 0.8813 |
| Platelet(×10^9^/L) |  | 185.0 [178.5,213.0] | 142.0 [128.5, 186.5] | 0.1868 | 165.0 [111.2, 223.8] | 192.0 [143.2,333.0] | 0.3732 |
| CRP (mg/L) |  | 29.6 [21.1, 49.7] | 92.9 [71.1, 105.1] | 0.0276 | 72.8 [22.8, 83.9] | 62.7 [25.9, 112.7] | 0.8053 |
| ALT (U/L) |  | 20.0 [17.0, 25.5] | 33.0 [15.0, 41.5] | 0.3931 | 32.0 [18.0, 36.8] | 31.0 [26.2, 54.2] | 0.5748 |
| AST (U/L) |  | 22.0 [21.0, 35.0] | 31.0 [27.0, 48.5] | 0.0487 | 39.0 [30.8, 45.8] | 32.0 [24.2, 55.0] | 1 |
| GGT (U/L) |  | 26.0 [20.5, 43.0] | 45.0 [26.0, 57.5] | 0.3085 | 26.0 [19.2, 40.0] | 56.0 [28.8, 132.2] | 0.1662 |
| TBIL (μmol/L) |  | 13.7 [13.0, 16.0] | 8.8 [8.4, 15.4] | 0.2119 | 14.6 [11.7, 19.9] | 12.6 [9.0, 20.1] | 0.6209 |
| DBIL (μmol/L) |  | 3.9 [3.5, 5.3] | 3.7 [3.1, 5.8] | 0.6917 | 5.2 [3.7, 8.2] | 4.8 [3.9, 6.6] | 0.7415 |
| Serum Creatinine (mmol/L) |  | 65.0 [58.6, 82.0] | 71.2 [62.9, 100.6] | 0.3928 | 68.4 [59.8, 75.2] | 69.8 [64.6, 87.5] | 0.7575 |
| Blood glucose (mmol/L) |  | 7.2[6.4, 10.6] | 6.6 [6.0, 8.0] | 0.2122 | 8.6 [6.7, 11.4] | 6.3 [5.6, 7.1] | 0.0411 |
| Duration of hospitalization (Days) |  | 9.0 [7.5, 10.0] | 19.0 [13.0, 23.5] | 0.0031 | 18.0 [9.5, 27.5] | 14.5 [11.8, 17.5] | 0.3392 |
| Hypertension (%) | 0 | 8 (72.73) | 5 (45.45) | 0.3872 | 5 (50.00) | 10 (83.33) | 0.1718 |
|  | 1 | 3 (27.27) | 6 (54.55) |  | 5 (50.00) | 2 (16.67) |  |
| Diabetes (%) | 0 | 10 (90.91) | 7 (63.64) | 0.3108 | 9 (90.00) | 12 (100.00) | 0.4545 |
|  | 1 | 1 (9.09) | 4 (36.36) |  | 1 (10.00) | 0 (0.00) |  |

Data are median (lQR) or n (%). 0=male or no; 1=female or yes; WBC= white blood cells; CRP=C-reactive protein; ALT=Alanine aminotransferase; AST=Aspartate aminotransferase;


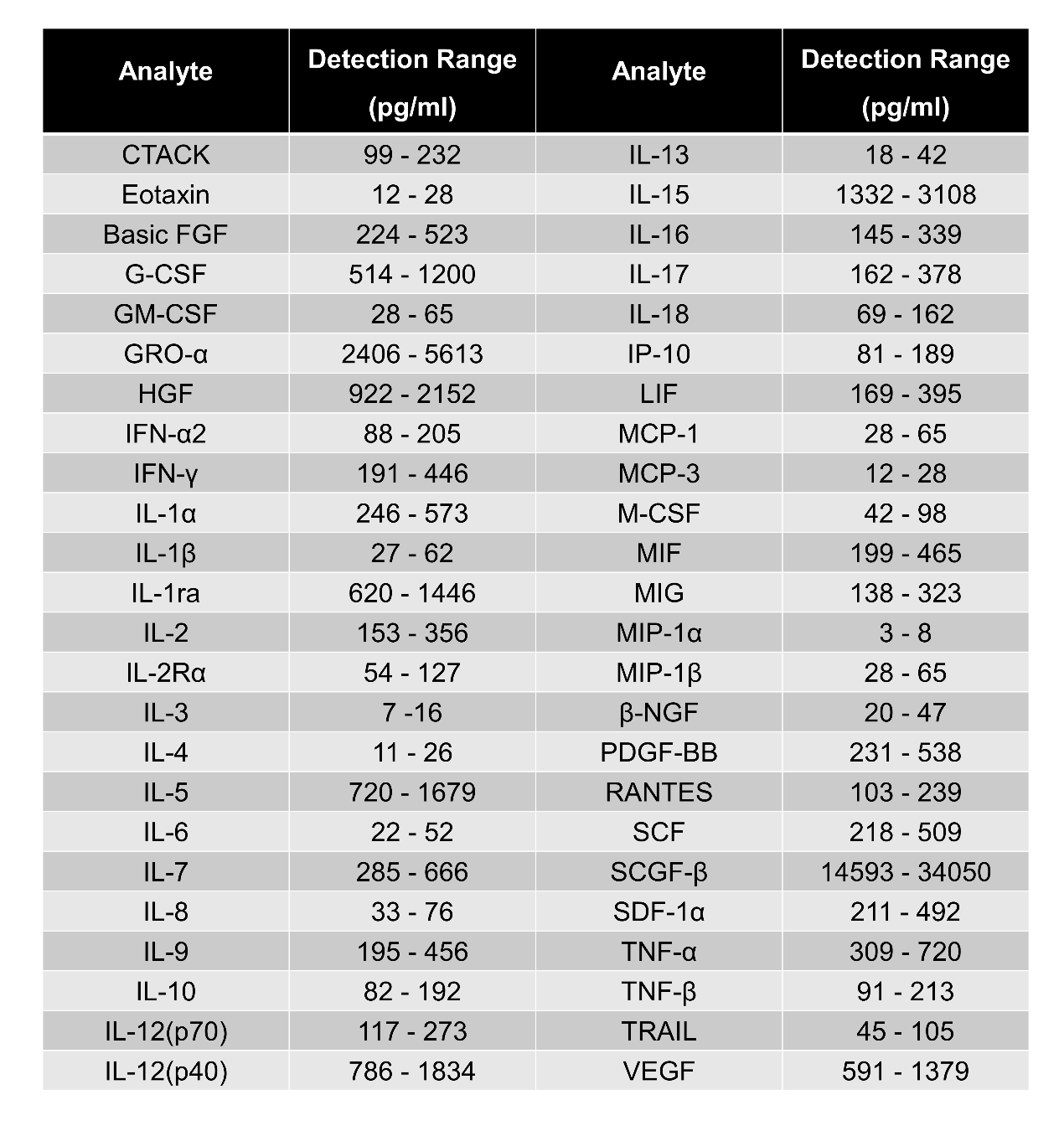


**Table S2. Detection ranges of 48 cytokines and chemokines**
